# Gastric inflammatory myofibroblastic tumor presented with severe anemia and inflammation: a case report

**DOI:** 10.1186/s40792-023-01802-9

**Published:** 2024-01-08

**Authors:** Sakura Hiramatsu, Ryo Ataka, Yusuke Nakayama, Miho Hirai, Ayako Hirata, Jun Takeshima, Kenjiro Hirai, Shinya Hamasu, Ikuo Aoyama, Tetsuro Hirose

**Affiliations:** 1 Department of Surgery, Otsu Red Cross Hospital, 1-1-35 Nagara, Otsu City, Shiga Japan; 2 Department of Gastroenterology, Otsu Red Cross Hospital, 1-1-35 Nagara, Otsu City, Shiga Japan

**Keywords:** Gut, Inflammatory myofibroblastic tumor, Anemia, Inflammation, Surgery

## Abstract

**Background:**

Inflammatory myofibroblastic tumor (IMT) is a rare stromal tumor, often found in children and young adults, and most commonly occurs in the lungs. Surgical resection is considered the standard treatment for localized IMT, although only limited data exist. Gastric IMT in adults is extremely rare, and there are no established guidelines for its treatment.

**Case presentation:**

A 69-year-old male presented with persistent fatigue and weakness. Laboratory examination revealed severe anemia and inflammation. Upper gastrointestinal endoscopy at admission revealed a 40-mm type I softish tumor in the lesser curvature of the gastric body, without apparent hemorrhage. Repeated biopsies, including partial resection with snare, failed to give a definitive diagnosis. Computed tomography (CT) revealed a massive lesion at the gastric body, protruding into the gastric lumen, which was consistent with the gastric tumor. After admission, the patient developed anemia refractory to frequent blood transfusions despite the absence of apparent gastrointestinal bleeding. In addition, the patient had recurrent fevers of 38 °C or higher, and persistent high inflammatory levels. Fluorodeoxyglucose-positron emission tomography (FDG-PET) CT 1 month after the first visit exhibited an increased FDG uptake in the gastric tumor. In addition, this CT scan revealed a rapid increase in tumor size to 75 mm. It was suspected that the undiagnosed gastric tumor caused these serious clinical symptoms, and he underwent distal gastrectomy and cholecystectomy. The gross image of the tumor showed an 80-mm cauliflower-like shape with a gelatinous texture. The histopathological diagnosis was IMT. The postoperative course was uneventful, and the patient’s symptoms subsided drastically, improving both anemia and systemic inflammation. The patient has shown no recurrence or relapse of the symptoms over one and a half years.

**Conclusions:**

In this case, the tumor resection finally enabled the diagnosis of IMT and resolved the clinical symptoms. Despite its predominantly benign morphological nature, some cases of IMT present clinically adverse courses. Surgical treatment may lead to its final diagnosis and improvement of clinical symptoms.

## Background

Inflammatory myofibroblastic tumor (IMT) is an uncommon stromal neoplasm of intermediate malignancy [1]. IMT is preferentially found in children and young adults, and most commonly occurs in the lungs. The initial symptoms depend on the primary tumor location. Surgical resection is considered the standard treatment for localized IMT, and there are limited data to support the use of radiation or systemic chemotherapy. Gastric IMT in adults is extremely rare, and there are no established guidelines for its treatment [1].

Herein, we present a case of a giant gastric tumor that showed severe anemia and high fever. We had difficulty in making its preoperative diagnosis and in dealing with anemia and inflammatory symptoms. Its surgical resection led to its definite diagnosis as gastric IMT and total recovery of the patient.

## Case presentation

**Fig. 1:**
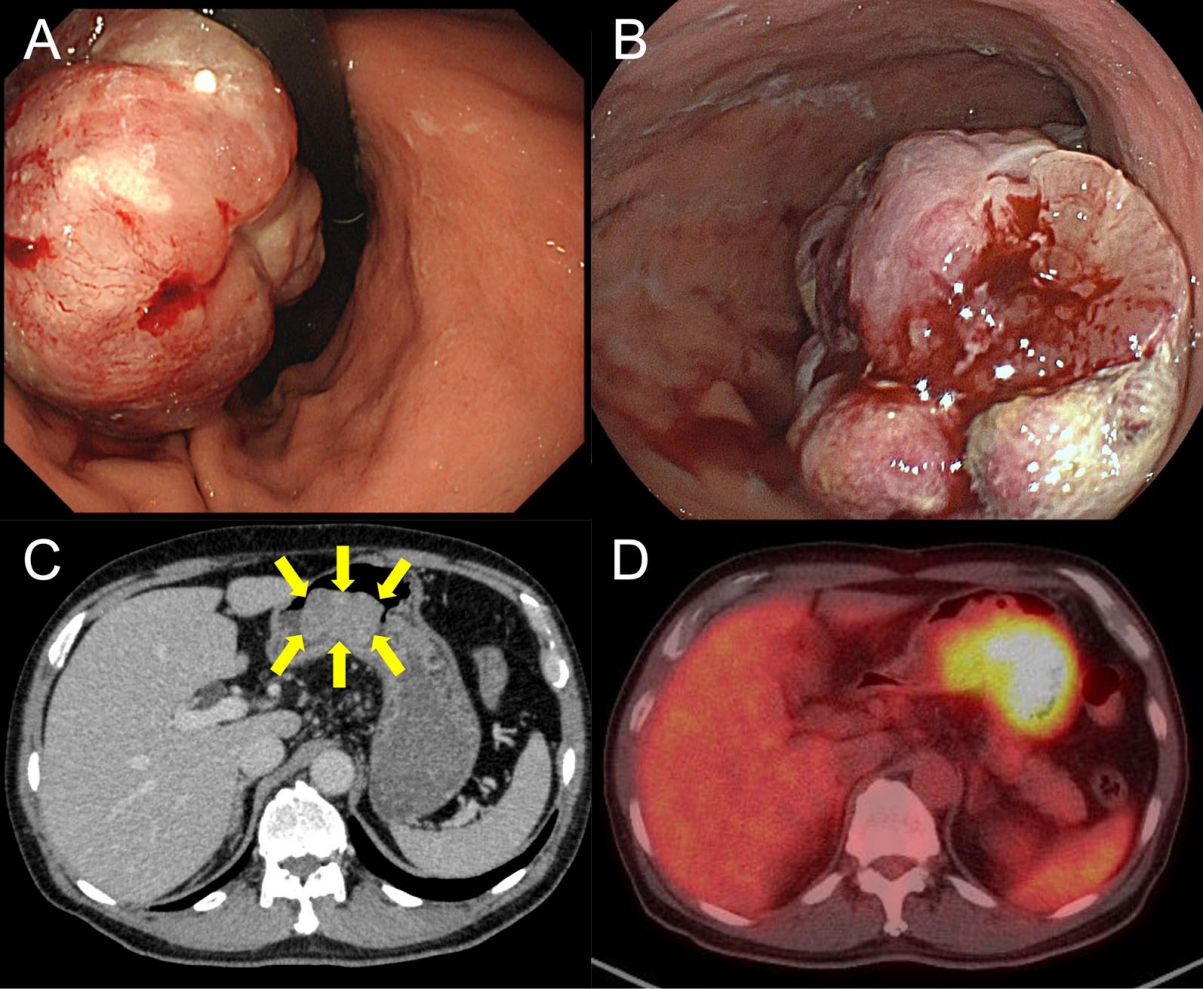
**A** Upper gastrointestinal endoscopy at admission revealed a 40-mm type I softish tumor in the lesser curvature of the gastric antrum, without apparent tumor hemorrhage. **B** Bloc biopsy was performed. The tumor section was gelatinous. **C** Contrast-enhanced computed tomography (CT) showed a massive lesion at the gastric body, protruding into the gastric lumen (arrows). **D** Fluorodeoxyglucose-positron emission tomography (FDG-PET) CT one month after the first visit exhibited an FDG uptake in the gastric body (maximum standardized uptake value 8)

**Fig. 2:**
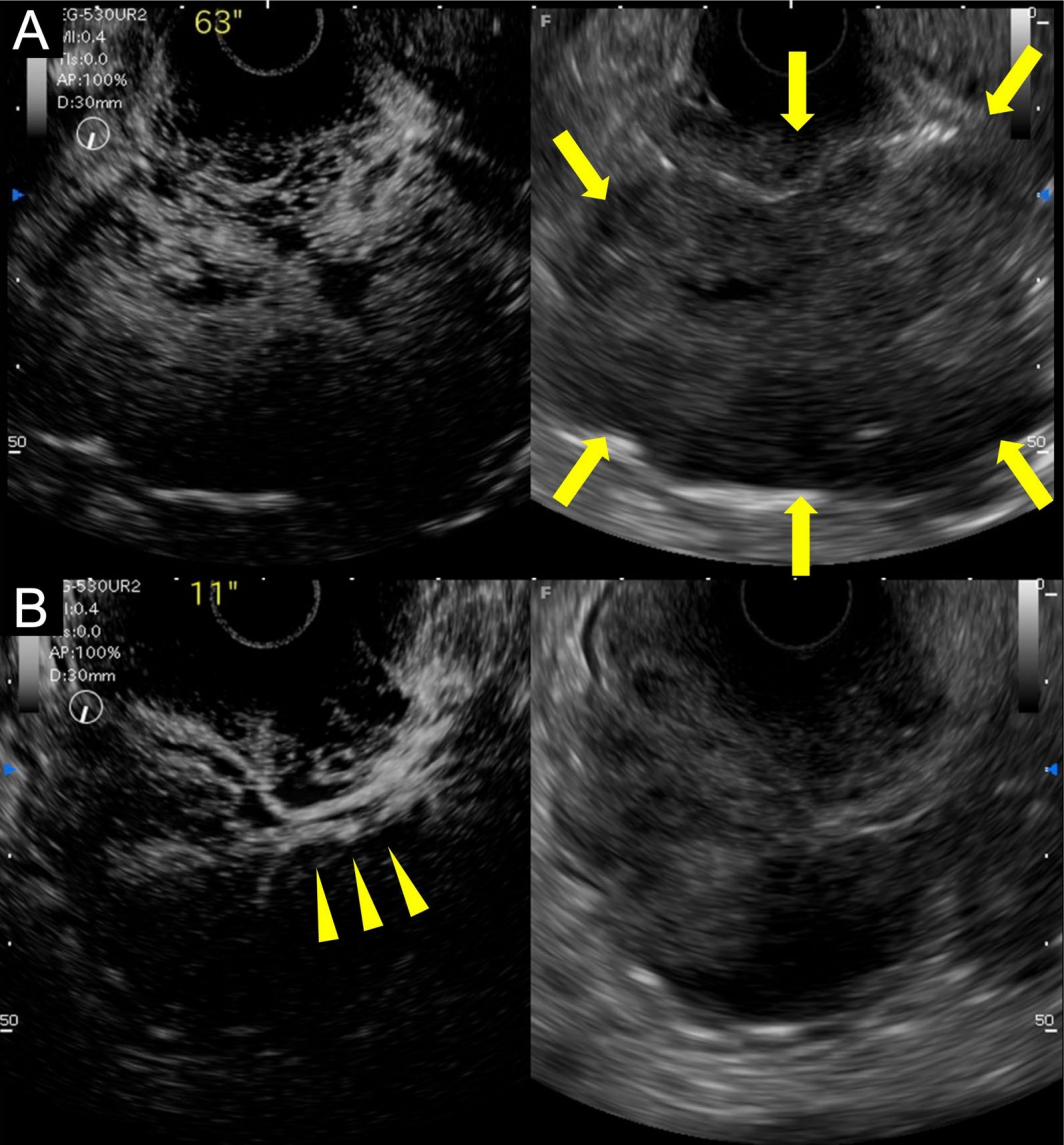
The left side is the contrast-applied image of the right-side image. **A** Endoscopic ultrasound showed heterogeneous internal echoes with partially mottled areas (arrows). **B** Applied contrast revealed rapid blood flow from the vessels at the stem (arrowheads), clarifying its internal structure

A 69-year-old Japanese male was referred to our hospital for persistent fatigue and weakness over the past few days. His past medical history was dyslipidemia, cholelithiasis, and gastric hyperacidity. Besides a high fever of 38.7 °C, his physical examination was unremarkable. Laboratory examination revealed very low hemoglobin (Hb) (5.8 g/dL), low hematocrit (19.2%), normal mean corpuscular volume (82.4 fL), and low mean corpuscular hemoglobin (24.9 pg), showing normocytic hypochromic anemia. Elevated C-reactive protein (CRP) (10 mg/dL), high platelet cell count (55.4 × 10^4^/μ), and low albumin level (2.7 g/dL) were also detected. Tumor markers were in the normal range (CA19-9, 3.4 U/mL; CEA, 1.0 ng/mL; AFP, 3.3 ng/mL). In addition, immunological tests ruled out infectious and autoimmune diseases. Antibiotics were administered, until the result of the blood culture revealed no growth of bacteria, and the high inflammation status did not improve. Upper gastrointestinal endoscopy at admission revealed a 40-mm softish tumor in the lesser curvature of the gastric body, without apparent hemorrhage. The tumor had a smooth shape and was overlaid by erosion covered with mucus (Fig. 1A). Its texture was so softish that biopsy forceps easily penetrated deep into the tumor. Repeated biopsies, including partial resection to obtain sufficient biopsy material, could obtain only mucous-rich granulation tissue with inflammation, confirming no definitive diagnosis (Fig. 1B). Contrast-enhanced computed tomography (CT) showed a massive lesion at the gastric body, protruding into the gastric lumen, which was consistent with the gastric tumor (Fig. 1C). Endoscopic ultrasound showed its semipeduncurated structure with heterogeneous internal echoes and partially mottled areas, although the tumor was too large to obtain its overview at the initial endoscopic observation. Applied contrast revealed rapid blood flow from the vessels at the stem, clarifying its internal structure (Fig. 2). After admission, the patient developed refractory anemia despite the absence of apparent gastrointestinal bleeding and required frequent blood transfusions. Serum iron was 12 μg/dL, and unsaturated iron binding capacity (UIBC) was 183 μg/dL, ferritin was 432.9 ng/mL. In addition, he had a recurrent fever over 38 °C and a high CRP level of around 20 mg/dL, suggesting severe inflammation. Fluorodeoxyglucose-positron emission tomography (FDG-PET) CT 1 month after the first visit exhibited an increased FDG uptake in the gastric tumor (maximum standardized uptake value 8), consistent with the tumor. In addition, this CT scan revealed a rapid increase in tumor size to 75 mm (Fig. 1D). We suspected that the undiagnosed gastric tumor caused these serious clinical symptoms and decided to perform its surgical resection. For the surgical procedure, we selected open distal gastrectomy. The reason we chose distal gastrectomy was that the tumor was too large to perform local resection. In addition, strong adhesion was expected due to severe inflammation, and high bleeding risk by intraoperative manipulation of the softish hypervascularized tumor was anticipated. Therefore, we decided to perform open surgery for this patient.

One month after the initial visit, the patient underwent distal gastrectomy and cholecystectomy with Roux-en-Y reconstruction. At laparotomy, the tumor was palpated in the lesser curvature of the gastric body, consistent with the preoperative image findings. Neither invasion to the adjacent organs nor metastasis to the peritoneum was observed. After resecting the duodenum just caudal to the pylorus, the stomach was dissected at the cranial side of the tumor. During this procedure, we paid careful attention not to cause massive bleeding by crushing the tumor. Also, we dissected its surrounding lymph nodes, according to our usual procedure of gastric carcinoma. The operation time was 328 min, and the blood loss volume was 175 ml.

**Fig. 3:**
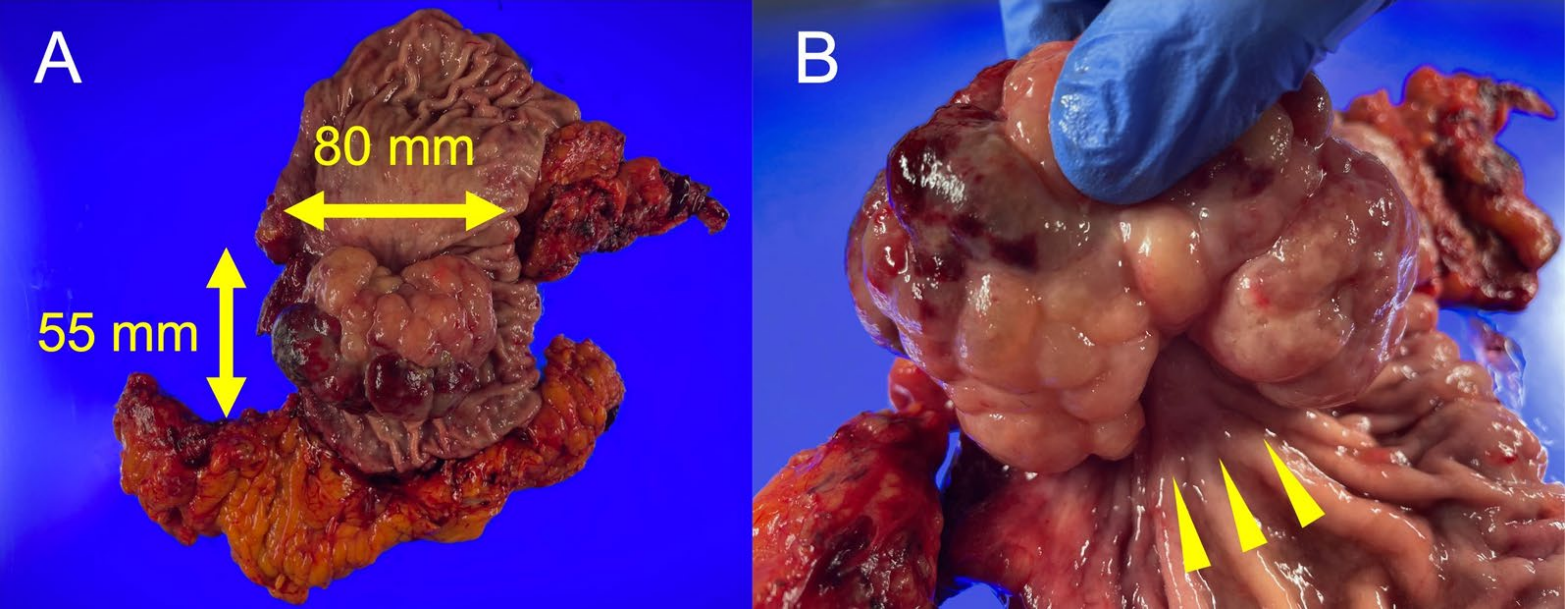
**A** The gross image of the tumor showed an 80-mm cauliflower-like shape with a gelatinous texture. **B** The stem of the semipeduncurated tumor (arrowheads)

**Fig. 4:**
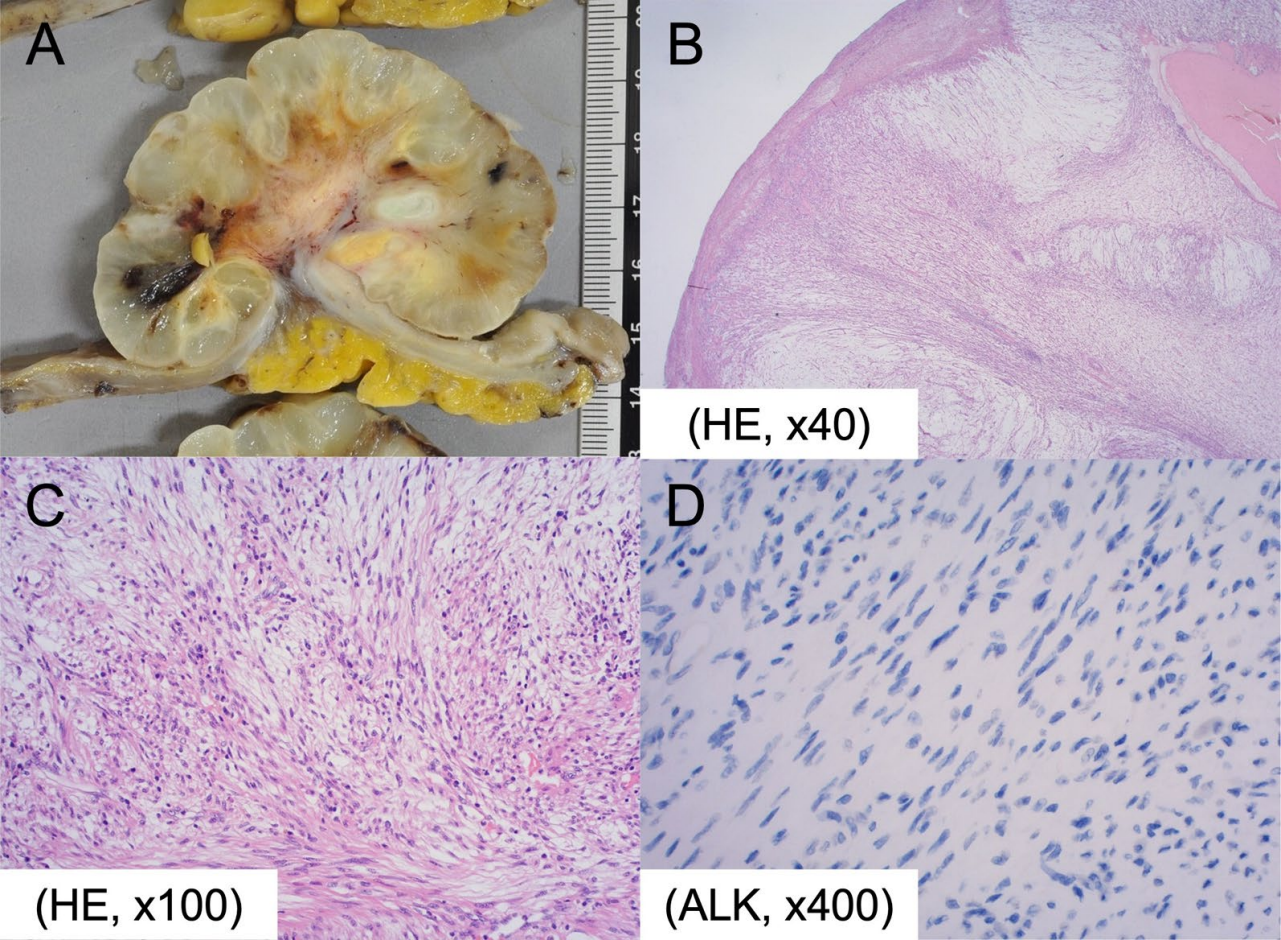
**A** The gross image of the tumor showed an 80-mm cauliflower-like shape with a gelatinous texture. **B** Histopathological examination demonstrated proliferation of spindle cells and collagen fiber, and the infiltration of plasma cells and lymphocytes (HE, × 40). **C** The myxoid change decreased toward the central region, and inflammatory cell infiltration, including neutrophils, increased instead (HE, × 100). **D** ALK expression was negative (ALK, × 400). *HE* hematoxylin eosin, *ALK* anaplastic lymphoma kinase

The gross image of the tumor showed an 80-mm cauliflower-like shape with a gelatinous texture (Fig. 3A, B). Histopathological diagnosis of the specimen revealed extensive mucous-type stromal degeneration at the outer region of the tumor, and predominant inflammatory cell infiltration in the central region, which led to the final diagnosis of IMT. The myxoid change decreased toward the central region, and inflammatory cell infiltration, including neutrophils, increased instead (Fig. 4A–C). No mitosis image was found in the proliferating cells. Immunohistochemical staining of anaplastic lymphoma kinase (ALK) showed negative ALK expression. Gastric regional lymph nodes showed no malignancy (Fig. 4D).

**Fig. 5:**
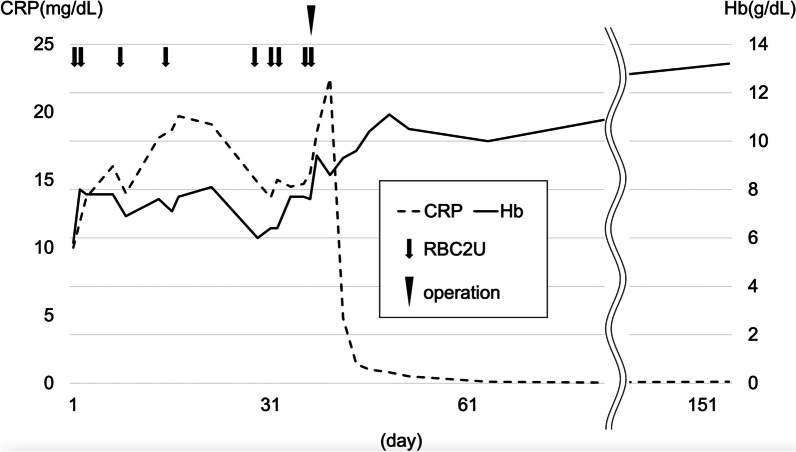
Clinical course of the present case. *CRP* C-reactive protein, *Hb* hemoglobin, *RBC2U*, transfusion of two units of packed red blood cells

The postoperative course was uneventful, and the patient’s symptoms subsided drastically, showing improved anemia and systemic inflammation. He was discharged on postoperative day 16 with normal laboratory data; Hb 11.1 g/dL and CRP 0.8 mg/dL. The patient has neither tumor recurrence nor relapse of the symptoms over one and a half years (Fig. 5).

## Discussion

IMT is a very rare mesenchymal neoplasm, with 150–200 new cases diagnosed annually in the United States [2]. It is of low to intermediate malignancy and rarely metastasizes. It tends to occur in children and young adults, with a mean age of 9–10 years. It has been reported that, in children IMT manifests with chromosomal abnormalities that include ALK gene rearrangement, whereas in older patients, it shows the proliferation of mesenchymal cells after inflammatory stimuli, often infectious stimuli. Therefore, pediatric IMT may result from a tumorous process, and adult IMT may originate from an inflammatory process [3]. IMT most commonly originates from the lung and can also arise in any site of the body (e.g., abdomen, pelvis, retroperitoneum, soft tissues, bone, larynx, uterus, liver, kidney, and central nervous system). The initial symptoms depend on the primary tumor location. Surgical resection is the standard treatment for localized IMT. There are limited data to support the use of radiation or systemic chemotherapy. The diagnosis of IMT is made by gross and histologic findings on biopsy. Gross image of IMT is often firm, fleshy, or gelatinous, with a white or yellowish cut surface. Its pathology is characterized by spindle cell proliferation and an inflammatory infiltrate. ALK gene mutation is found in ~ 50% of IMT.

Gastric IMT in adults is extremely rare. There have been only 26 reported cases of adult gastric IMT since 2000 (Table 1) [4–26]. The mean age of the patients was 42.6-year old. Males accounted for 12 cases (46.2%) and females for 14 cases (53.8%). The most common symptom was abdominal pain (15 cases, 53.8%), followed by anemia (10 cases, 38.4%). Other symptoms included malaise, fever, weight loss, and the presence of a palpable mass, but some asymptomatic cases were found by chance during routine medical checkups. The tumor was most commonly observed in the middle third of the stomach (13 cases, 50%). The mean tumor size of the 26 cases was 7.35 cm. The most common surgical treatments were distal gastrectomy (5 cases, 19.2%) and partial gastrectomy (5 cases, 19.2%). Immunohistochemical staining showed positive for ALK in 11 cases (42.3%). Muscle invasion was seen in more than half of the cases (14 cases, 53.8%). Lymph node metastasis was observed in 2 cases (7.7%). Recurrence was seen in 2 patients (7.7%); one was local recurrence and the other was peritoneal seeding.

**Table 1:** 26 adult gastric IMT cases since 2000

Age (year old) (mean [range])	42.6 [18–68]
Sex	
Male	12 (46.2%)
Female	14 (53.8%)
Symptom	
Abdominal pain	15 (53.8%)
Anemia	10 (38.4%)
Weight loss	6 (23.1%)
Inflammation	5 (19.2%)
Abdominal mass	4 (15.3%)
Nausea/vomiting	4 (15.3%)
Abdominal distention	3 (11.5%)
Fever	1 (3.8%)
Hematemesis	1 (3.8%)
Dysphagia	1 (3.8%)
Acid reflux	1 (3.8%)
Night sweating	1 (3.8%)
Asymptomatic	1 (3.8%)
Unknown	3 (11.5%)
Tumor location	
Upper third	4 (15.4%)
Middle third	13 (50%)
Lower third	9 (35.6%)
Tumor size (cm) (mean [range])	7.35 [2–22]
Surgery	
Distal gastrectomy	5 (19.2%)
Proximal gastrectomy	1 (3.8%)
Total gastrectomy	4 (15.3%)
Partial gastrectomy	5 (19.2%)
Bypass	1 (3.8%)
Endoscopic mucosal resection	1 (3.8%)
Unknown	9 (34.6%)
Anaplastic lymphoma kinase	
Positive	11 (42.3%)
Negative	9 (34.6%)
Not available	6 (23.1%)
Muscle invasion	14 (53.8%)
Lymph node metastasis	2 (7.7%)
Recurrence	
Local recurrence	1 (3.8%)
Peritoneal seeding	1 (3.8%)

One of the challenges associated with IMT is the difficulty of preoperative diagnosis. Although biopsy is the best method to diagnose IMT, none of the above 26 cases had a preoperative diagnosis of IMT. The preoperative diagnoses in these previous reports included malignant tumors, such as gastric carcinoma, sarcoma, and gastrointestinal stromal tumor. In our case, multiple biopsies failed to confirm the diagnosis as well. This was presumably because of the intensive myxoid change on the exterior of the tumor. The outer myxoid change was so thick that preoperative biopsy could not determine the final diagnosis.

Another problem of IMT is that, despite its less malignant nature, it can have a clinically adverse course. One of the major problems is refractory anemia. In primary gastric IMT, it is difficult to determine whether anemia is caused by gastrointestinal hemorrhage or chronic inflammation. In our case, severe anemia, high inflammation and excessive growth rate led to the clinically adverse course. Despite the absence of obvious gastrointestinal bleeding, the patient developed refractory anemia. Also, the patient had a recurrent fever of 38 °C or higher and persistent high inflammatory levels. Given its abnormal situation and laboratory data, we believe that anemia correlated with inflammatory findings. Moreover, the tumor enlarged extremely fast in our case, where the tumor size increased from 40 to 75 mm in one month.

Surgical tumor resection is the optimal treatment for primary gastric IMT, and it results in total recovery from severe symptoms, as shown in the present case. The standard surgical procedure is en bloc resection with R0 margins. Depending on tumor size, the surgical approach could be wide local excision involving removal of the tumor with some surrounding normal tissue to ensure complete excision [1]. In more than half of the 26 cases, tumors invaded the muscle layer. Therefore, although EMR was selected as the surgical procedure in one case, we consider whole-layer resection to be the first choice. Also, lymph node metastasis or recurrence was observed in some cases. Therefore, we considered gastrectomy with lymph node dissection can be acceptable for gastric IMT. In fact, because it is almost impossible to make the preoperative diagnosis of IMT as shown above, we recommend considering the possibility of a malignant tumor when making its perioperative decisions.

## Conclusions

We have presented a case of IMT, in which the tumor resection finally led to the diagnosis of IMT and resolved the clinical symptoms. Gastric IMT is an extremely rare stromal tumor. Despite its predominantly benign morphological nature, some cases of IMT present clinically adverse courses, as shown in this case. Surgical treatment may lead to its final diagnosis and improve its clinical symptoms.

## Data Availability

Not applicable.

## Abbreviations

ALP: Anaplastic lymphoma kinase
CRP: C-reactive protein
CT: Computed tomography
FDG: Fluorodeoxyglucose
Hb: Hemoglobin
HE: Hematoxylin eosin
IMT: Inflammatory myofibroblastic tumor
PET: Positron emission tomography
UIBC: Unsaturated iron binding capacity
